# The Efficacy of Flogofilm^®^ in the Treatment of Chronic Bacterial Prostatitis as an Adjuvant to Antibiotic Therapy: A Randomized Prospective Trial

**DOI:** 10.3390/jcm12082784

**Published:** 2023-04-09

**Authors:** Biagio Barone, Benito Fabio Mirto, Alfonso Falcone, Francesco Del Giudice, Achille Aveta, Luigi Napolitano, Dario Del Biondo, Matteo Ferro, Gian Maria Busetto, Celeste Manfredi, Daniela Terracciano, Raffaele Gambardella, Savio Domenico Pandolfo, Francesco Trama, Ciro De Luca, Raffaele Martino, Federico Capone, Gaetano Giampaglia, Enrico Sicignano, Octavian Sabin Tataru, Giuseppe Lucarelli, Felice Crocetto

**Affiliations:** 1 Department of Neurosciences and Reproductive Sciences and Odontostomatology, University of Naples “Federico II”, 80131 Naples, Italy; 2 Department of Maternal Infant and Urologic Sciences, Policlinico Umberto I Hospital, “Sapienza” University of Rome, 00161 Rome, Italy; 3 Department of Urology, Ospedale del Mare, ASL NA1 Centro, 80147 Naples, Italy; 4 Department of Urology, European Institute of Oncology (IEO) IRCCS, 20141 Milan, Italy; 5 Department of Oncology and Hemato-Oncology, University of Milan, 20141 Milan, Italy; 6 Department of Urology and Organ Transplantation, University of Foggia, 71122 Foggia, Italy; 7 Urology Unit, Department of Woman, Child and General and Specialized Surgery, University of Campania “Luigi Vanvitelli”, 80131 Naples, Italy; 8 Department of Translational Medical Sciences, University of Naples Federico II, 80131 Naples, Italy; 9 Unit of Urology, ASL NA1 Centro, 80145 Naples, Italy; 10 Department of Simulation Applied in Medicine, The Institution Organizing University Doctoral Studies (I.O.S.U.D.), George Emil Palade University of Medicine, Pharmacy, Sciences, and Technology from Târgu Mureș, 540142 Târgu Mureș, Romania; 11 Urology, Andrology and Kidney Transplantation Unit, Department of Precision and Regenerative Medicine and Ionian Area, University of Bari “Aldo Moro”, 70124 Bari, Italy

**Keywords:** chronic prostatitis, fluoroquinolones, NIH-CPSI, IPSS, Flogofilm^®^

## Abstract

Introduction: Bacterial prostatitis (BP) is a common prostatic infection characterized by a bimodal distribution in young and older men, with a prevalence between 5–10% among all cases of prostatitis and a high impact on quality of life. Although the management of bacterial prostatitis involves the use of appropriate spectrum antibiotics, which represent the first choice of treatment, a multimodal approach encompassing antibiotics and nutraceutical products in order to improve the efficacy of chosen antimicrobial regimen is often required. Objective: To evaluate the efficacy of Flogofilm^®^ in association with fluoroquinolones in patients with chronic bacterial prostatitis (CBP). Methods: Patients diagnosed with prostatitis (positivity to Meares–Stamey Test and symptoms duration > 3 months) at the University of Naples “Federico II”, Italy, from July 2021 to December 2021, were included in this study. All patients underwent bacterial cultures and trans-rectal ultrasounds. Patients were randomized into two groups (A and B) receiving antibiotic alone or an association of antibiotics plus Flogofilm^®^ tablets containing Flogomicina^®^ for one month, respectively. The NIH-CPSI and IPSS questionnaires were administered at baseline, four weeks, twelve and twenty-four weeks. Results: A total of 96 (Group A = 47, Group B = 49) patients concluded the study protocol. The mean age was comparable, with a mean age of 34.62 ± 9.04 years for Group A and 35.29 ± 10.32 years for Group B (*p* = 0.755), and IPSS at the baseline was 8.28 ± 6.33 and 9.88 ± 6.89 (*p* = 0.256), respectively, while NIH-CPSI at baseline was 21.70 ± 4.38 and 21.67 ± 6.06 (*p* = 0.959), respectively. At 1, 3 and 6 months, the IPSS score was 6.45 ± 4.8 versus 4.31 ± 4.35 (*p* = 0.020), 5.32 ± 4.63 versus 3.20 ± 3.05 (*p* = 0.042) and 4.91 ± 4.47 versus 2.63 ± 3.28 (*p* = 0.005) for Groups A and B, respectively. Similarly, the NIH-CPSI total score at 1, 3 and 6 months was 16.15 ± 3.31 versus 13.10 ± 5.03 (*p* < 0.0001), 13.47 ± 3.07 versus 9.65 ± 4.23 (*p* < 0.0001) and 9.83 ± 2.53 versus 5.51 ± 2.84 (*p* < 0.0001), respectively. Conclusions: Flogofilm^®^, associated with fluoroquinolones, demonstrate a significant improvement in pain, urinary symptoms and quality of life in patients affected by chronic bacterial prostatitis in both IPSS and NIH-CPSI scores compared with fluoroquinolones alone.

## 1. Introduction

Bacterial prostatitis (BP) represents one of the most important diseases worldwide, with a prevalence of 5–10% of all prostatitis cases, and the first reason to consult urologists among young men. According to the National Institute of Diabetes Digestive and Kidney Diseases of the National Institutes of Health (NIDDK/NIH), prostatitis is classified as acute bacterial prostatitis (category I), chronic bacterial prostatitis (category II), chronic prostatitis inflammatory/chronic pelvic pain syndrome-CP/CPPS (category IIIa), chronic prostatitis non-inflammatory/chronic pelvic pain syndrome-CP/CPPS (category IIIb) and asymptomatic inflammatory prostatitis (category IV). The mainstay of acute bacterial prostatitis and chronic bacterial prostatitis is the use of proper antibiotic treatment. In particular, acute bacterial prostatitis is treated according to the recommendation for complicated urinary tract infections, i.e., cephalosporins or fluoroquinolones, while chronic bacterial prostatitis has the use of fluoroquinolones as first-line treatment [1]. Several therapies, as well as antibiotics, anti-inflammatory medications and alpha-blockers, have been proposed. In particular, phytotherapy and physical therapy represent a safety and efficacy option, mostly in the era of antibiotic resistance [2,3,4]. The use of plant extracts as therapeutic agents is widely used in several urological diseases [5,6,7,8,9]. These alternative therapies show low adverse effects, high efficacy and potency and improve the efficacy of other therapies [10]. Generally, phytotherapy is associated with antibiotic treatment to improve their efficacy and reduce their use [11,12]. In the last years, several plant extracts alone or in combination have been proposed in prostatitis management and have reported a continuous increase in the number of available products. The basis for the use of these products relies upon the observations regarding the effects and roles of different foods on the prostate gland. The Western diet, characterized by a high intake of red processed meats and fats, has been associated with several diseases and disturbances in the urological field [5,13,14,15]. Conversely, the Mediterranean diet, characterized by a large use of olive oil, fibers, fish, fruits and vegetables—foods rich in compounds with antioxidant properties—represents a protective factor regarding prostatitis and prostate cancer [2,16,17]. Several studies have indeed demonstrated the marked anti-inflammatory effect of Serenoa Repens, an extract from the Saw palmetto, on the prostate gland, while quercetin, a natural flavonoid found in several fruits and vegetables, has similarly reported antioxidant and anti-inflammatory properties in animal and human studies [18,19,20]. Here, we focused our attention on a new original nutraceutical formulation called Flogofilm^®^. The aim of the present study was to evaluate the efficacy of an association therapy with fluoroquinolones and nutraceutical supplements for the treatment of patients with CBP.

## 2. Materials and Methods

We designed a prospective, non-blinded, randomized controlled trial (RCT) (registration code 429/21) enrolling 100 consecutive patients with CBP attending the Urology Clinic of the University of Naples “Federico II” from July 2021 to December 2021. This study was conducted in accordance with the Declaration of Helsinki and Good Clinical Practice (GCP) guidelines. Written informed consent was obtained from all participants involved in this study. The study protocol was approved by the University of Naples “Federico II” ethical review board via document number 429/21. CBP diagnosis was made in accordance with the definition of the disease: symptoms duration > 3 months, including dysuria, pelvic pain and/or discomfort plus positivity to the Meares–Stamey test [21]. Inclusion criteria were the following: patient age between 18 and 50 years, symptoms suggesting CBP, presence of prostatic calcification at a transrectal ultrasound and a positive Mears–Stamey test. Exclusion criteria of the study were the following: age less than 18 years, history of neurological disease, urinary stones or cancer, allergy to fluoroquinolones or other components of Flogofilm^®^, post-void residual > 50 mL, treatment with an alpha-blocker or 5-ARI, prostatic surgery, antibiotic treatments in the last four weeks, refusal to sign the informed consent and incomplete follow-up time [22]. The bacteria considered were only uropathogens: enteric gram-negative rods, enterococci, *Staphylococcus saprophyticus* and group B streptococci. All patients who tested positive for *Chlamydia trachomatis* (Ct), *Ureaplasma urealyticum*, *Neisseria gonorrhoeae*, herpes simplex viruses (HSV 1/2) and human papillomavirus (HPV) were also excluded. 

Patients were randomized 1:1 via simple randomization utilizing desktop software to generate a random sequence of numbers. According to the group, the treatment schedule was based on oral fluoroquinolone alone (levofloxacin, 1 tablet 500 mg daily for 4 weeks) (Group A) or fluoroquinolone plus 2 tablets of Flogofilm^®^ bis in die for 4 weeks (Group B). The Meares–Stamey test was repeated 1 month after therapy. All eligible patients underwent a thorough medical history, urological examination, uroflowmetry, Mears–Stamey test, PSA evaluation and transrectal ultrasound after signing an informed consent to participate in this study. In addition, patients compiled the National Institutes of Health Chronic Prostatitis Symptom Index (NIH-CPSI) and the International Prostatic Symptoms Score (IPSS) questionnaires (Italian versions). A follow-up examination was scheduled at 1, 3 and 6 months from the start of the therapy, and each patient underwent the same questionnaires compiled at the beginning of the treatment. 

### 2.1. Composition and Characterization of Flogofilm^®^

Flogofilm^®^ is a phytotherapeutic compound constituted by NAC (300 mg), bromelin (200 mg), vitamin C (100 mg), Ribes nigrum (100 mg), resveratrol (50 mg) and pelargonium (45 mg).

### 2.2. Statistical Analysis

Descriptive statistics were reported as means and standard deviations for continuous variables, while frequencies and percentages were reported for categorical variables. The normality of data was assessed via the Kolmogorov–Smirnov test. Sample size calculation was based on the IPSS score, considering alpha = 0.05, power = 80% and anticipated means of Groups A and B = 8 ± 3.5 and 6 ± 1.5. The final sample size = 94. Comparisons among groups were performed using the independent-sample Mann–Whitney U test for continuous variables and the Chi-square test for categorical variables. Statistical analysis was conducted using IBM SPSS software (version 25, IBM Corp., Armonk, NY, USA), considering *p* < 0.05 as statistically significant.

## 3. Results

**Figure 1:**
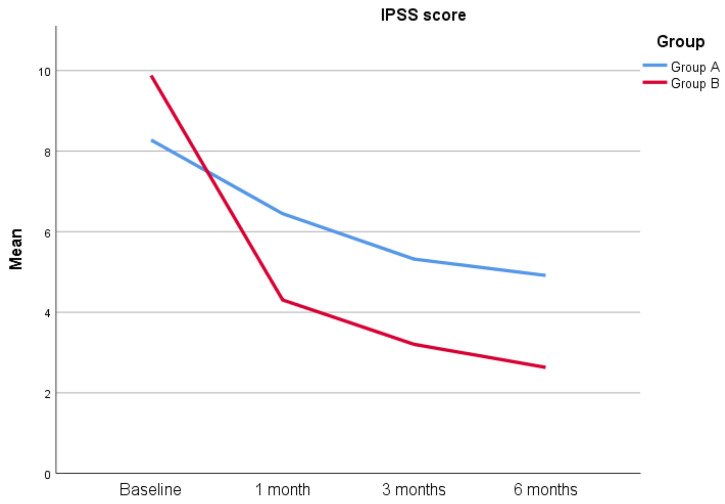
IPSS score from baseline to 6 months after treatment.

**Figure 2:**
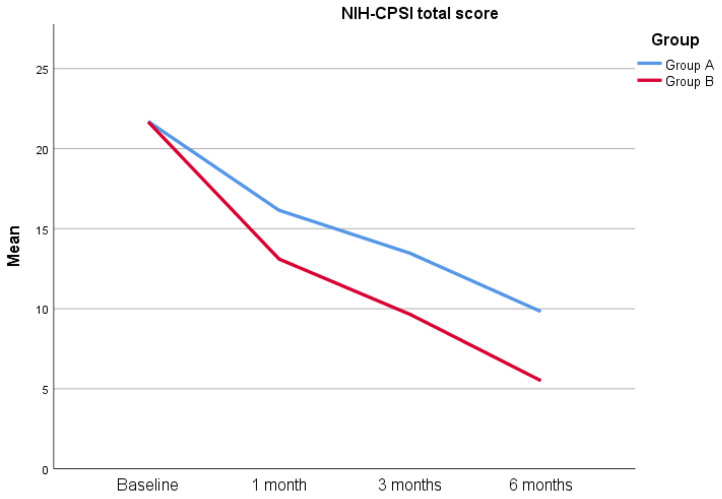
NIH-CPSI total score from baseline to 6 months after treatment.

A total of 100 patients were included in this study according to the eligibility criteria, and four patients were lost at the follow-up, yielding a total of 96 patients included in this study (47 in Group A and 49 in Group B). The most common bacteria found were *Escherichia coli* (83%), *Enterococcus faecalis* (10%), *Proteus mirabilis* (5%) and *Klebsiella aerogenes* (2%). The descriptive statistics of the cohort involved are reported in Table 1. As reported in the table, both groups are comparable in terms of age (34.62 ± 9.04 years versus 35.29 ± 10.32 years) (*p* = 0.755), serum PSA levels (1.14 ± 0.67 ng/mL versus 1.25 ± 0.6 ng/mL) (*p* = 0.265), prostate volume (31.51 ± 8.07 mL versus 31.10 ± 8.73 mL) (*p* = 0.962) and Qmed (20.89 ± 1.52 mL/s versus 21.41 ± 1.66 mL/s) (*p* = 0.071). Additionally, the IPSS score (8.28 ± 6.33 versus 9.88 ± 6.89) (*p* = 0.256), as well as the NIH-CPSI total score (21.70 ± 4.38 versus 21.67 ± 6.06) (*p* = 0.959) and relative subsets, are comparable at the beginning of the study (Table 1). No patients reported a positive Meares–Stamey test after 1 month. The IPSS score at 1 month is 6.45 ± 4.8 for Group A versus 4.31 ± 4.35 for Group B (*p* = 0.020), while the NIH-CPSI total score is 16.15 ± 3.31 versus 13.10 ± 5.03 (*p* < 0.0001), respectively. When analyzing the NIH-CPSI subsets, the pain domain reports a score of 5.83 ± 2.57 (Group A) versus 5.04 ± 4.66 (Group B) (*p* = 0.011), while urinary and quality of life (QoL) domains report a score of 4.89 ± 1.38 versus 3.76 ± 1.33 (*p* < 0.0001) and 5.43 ± 1.26 versus 4.31 ± 1.23 (*p* < 0.0001). The IPSS score at 3 months is 5.32 ± 4.63 for Group A versus 3.20 ± 3.05 for Group B (*p* = 0.042), while, similarly, the NIH-CPSI total score is 13.47 ± 3.07 versus 9.65 ± 4.23 (*p* < 0.0001), respectively. The NIH-CPSI pain domain at 3 months is 4.55 ± 2.54 (Group A) versus 3.18 ± 4.13 (Group B) (*p* < 0.0001), while the NIH-CPSI urinary and QoL domains at 3 months are 4.23 ± 1.11 versus 3.51 ± 1.24 (*p* = 0.004) and 4.68 ± 0.98 versus 2.96 ± 1.24 (*p* < 0.0001), respectively. Finally, the IPSS score at 6 months is 4.91 ± 4.47 for Group A versus 2.63 ± 3.28 (*p* = 0.005) for Group B, while the NIH-CPSI total score is 9.83 ± 2.53 versus 5.51 ± 2.84 (*p* < 0.0001), respectively. The NIH-CPSI pain domain at 6 months is 3.09 ± 2.12 for Group A versus 1.31 ± 2.13 for Group B (*p* < 0.0001), while the urinary domain is 3.02 ± 1.03 versus 2.43 ± 1.06 (*p* = 0.005), respectively. Finally, the QoL domain is 3.72 ± 1.25 versus 1.78 ± 1.34 (*p* < 0.0001) (Figure 1) (Figure 2) (Table 2).

## 4. Discussion 

Patients with chronic prostatitis represent a common and wide challenge in urological practice [23,24,25]. Although conventional therapies and antibiotic therapy are the mainstays of treating this condition, a proportion of patients do not respond to treatment despite reporting negative prostatic fluid cultures [26]. It has been proposed that chronic prostatitis may represent an inflammatory dysregulation in response to persistent chemokine upregulation, oxidant stress and cellular injury [27,28]. As long as an initial diagnosis of chronic prostatitis is established, it is evident that the condition is not a single disease entity, as different factors may be involved even in a single patient [29,30]. The use of antibacterial agents may not achieve the complete eradication of the infection due to multifactorial reasons, such as the poor distribution of drugs to the prostatic tissue, inadequate duration of antibiotic therapy or chemical modification in situ, which could impair the efficacy of the delivered treatment [31]. In particular, the formation of bacterial biofilm, i.e., a structure formed by microbial communities attached with substratum and embedded in a self-produced non-crystalline extracellular polymeric matrix, further impairs the efficacy of antibiotic therapy due to the protection provided by this structure to microbial communities against the antibiotic itself and the immune cells, in addition, to provide a biological niche, which permits the recurrence of the infection [32]. The use of phytotherapy in the treatment of chronic prostatitis has gained increased popularity due to the unique mechanism of action, low side-effect profiles and a high level of acceptance by the patient [33]. As reported in other studies, the possibility of using naturally extracted compounds in association with antibiotic therapy could improve the efficacy in terms of symptom relief and reduction of recurrences. To this regard, Cai et al. reported the efficacy of Serenoa Repens, quercetin and curcumin in association with prulifloxacin in patients affected by chronic bacterial prostatitis in terms of recurrence and improvement in quality of life, assessed via NIH-CPSI and IPSS score [34]. Similarly, Busetto et al. reported how short-lasting antibiotic therapy, in association with nutritional supplement constituted by Serenoa Repens and lactobacillus sporogens plus arbutin, showed better control and recurrence rate in patients affected by chronic bacterial prostatitis compared with antibiotic treatment alone [35]. Finally, more recently, Manfredi et al., in an analogous study comparing the association of nutritional supplement and antibiotic therapy versus antibiotic therapy alone, reported a significantly lower NIH-CPSI score and recurrence rate compared with those undergoing antibiotic therapy only [36]. The rationale related to our results also relies on the different compounds which constitute Flogofilm^®^.

*N*-Acetylcysteine (NAC) is a thiol-based antioxidant with antibacterial as well as antibiofilm, proteolytic and anti-adherence effects in vitro against several pathogens [37]. NAC plays a key role in enhancing the effect of co-administered antibiotics showing an antibiofilm activity with ciprofloxacin against *P. aeruginosa* [38]. In fact, according to a previously published study, NAC showed an intrinsic antimicrobial activity due to perturbation of the intracellular redox equilibrium and antibiofilm activity [39]. In the last case, NAC disrupts the structure of bacterial biofilm and improves the penetration of antibiotics. Nowadays, it is known that several bacteria involved in the development of acute and chronic prostatitis form biofilms [40]. This environment increases resistance to antibiotics, and the extracellular matrix protects the interior of the community. Bartoletti et al. showed that biofilm-producing bacteria were commonly involved in BP with a negative impact on the response to antibiotic therapy [41]. In addition, several studies reported polyphenols, tocopherols, phenolic acids, ascorbic acid and flavonoids. Polyphenols have been associated in vitro with strong antibacterial, antiviral, antimutagenic, anticancer, anti-inflammatory and antioxidant properties, including scavenging of free radicals, inhibition of lipid oxidation and a reduction of hydroperoxide formation [42]. Ribes nigrum is a well-recognized source of polyphenols, as well as anthocyanins, phenolic acid derivatives and proanthocyanidins. Ikuta et al. reported the antimicrobial and anti-inflammation activities of the Ribes nigrum, in particular, due to its capacity to prevent the adsorption and growth of bacteria [43].

Resveratrol is a non-flavonoid polyphenol compound with several biological properties, and it is widely used. Qian et al. reported that, in a murine model, resveratrol treatment attenuates the prostatic inflammation and downregulates the expression of IL-6, IL-8 and TNF-α in rats with chronic prostatitis [44]. In a recent publication, Wang et al. [45] reported that a combination of colistin and resveratrol could synergistically damage the bacterial cell membrane, inducing lysis and reducing drug resistance, and it could be a valid alternative to prevent colistin-resistance, particularly in a *P. aeruginosa* infection. Moreover, resveratrol is also able to modulate the composition and metabolite production of intestinal microbiota, sometimes involved in BP as a bacterial reservoir [46]. In this study, we showed that the combination therapy of Flogofilm^®^ plus fluoroquinolones significantly improved pain, urinary symptoms and quality of life in patients affected by chronic bacterial prostatitis compared to fluoroquinolones alone. In particular, both the IPSS score and NIH-CPSI total score reported a marked decrease in Group B compared with Group A since the first month, yielding, overall, a mean of −2 points in scores between both groups. Nevertheless, when analyzing the single domains of the NIH-CPSI score, a few interesting results are reported. The urinary domain score, as well as the quality of life score, reported a similar significant difference between the groups treated with fluoroquinolones alone versus fluoroquinolones plus Flogofilm^®^, with a particularly marked difference after 6 months. Pain domain scores showed instead, despite a statistically significant difference between the two groups, a narrow delta between the scores of the two groups, being fairly comparable in the first month after treatment. Despite the controversial data, our findings could be explained with the duration of pain in chronic prostatitis, which could last, albeit the therapy, up to three months. The reasons for this pain duration in chronic prostatitis, even after treatment, are multifactorial. Neuropathic inflammation, voiding patterns and psychological aspects are indeed considered in the overall evaluation of symptoms of the affected patient [47,48]. Lastly, it has to be considered that the Meares–Stamey test performed after 1 month could have influenced the pain score, at least psychologically. Due to these considerations, the effects of Flogofilm^®^ on the NIH-CPSI quality of life and urinary domains could be the main reason for better scores in the total NIH-CPSI score of Group B, highlighting the efficacy of Flogofilm^®^ in relieving the voiding symptoms in the first month after therapy. At the present time, to the best of our knowledge, there are no studies available about the use of Flogofilm^®^ in chronic BP management. We hypothesized that the clinically significant improvement observed in patients treated with Flogofilm^®^ was due to the particular characteristics of the different products. Our data were consistent with other similar studies reported in the literature. In particular, Busetto et al., in a study utilizing fluoroquinolones plus an association of saw palmetto extract, lactobacillus sporogens and arbuting versus antibiotic therapy alone, reported better NIH-CPSI scores for the first group compared to the second one. Differently from our study, effects on pain score were more marked since the first follow-up (6.20 ± 2.35 versus 2.13 ± 1.90 at 2 months), albeit considering the scores at 6 months of the control group (8.06 ± 2.81 versus 1.25 ± 0.99), this difference could be related to a potential selection bias. In addition, our cohort reported lower pain scores at the baseline compared to the cohort of Busetto et al. (9.62 ± 3.98 and 8.90 ± 5.18 compared to 11.65 ± 3.23 and 12 ± 2.78, respectively), while baseline urinary scores were higher in our study (5.66 ± 1.82 and 6.08 ± 1.6 versus 4.65 ± 2.11 and 4.26 ± 2.28) [35]. As a result, the difference in effects in pain scores could be related to the baseline characteristics of patients involved. Similarly, Cai et al. reported in an analogous study involving two groups treated with an antibiotic plus Serenoa Repens, urtica dioica, curcumin and quercitin, or antibiotic alone, had marked effects of antibiotic plus a nutraceutical product on NIH-CPSI total scores and IPSS scores. Additionally, in this case, the rapid efficacy of the treatment on the scores could be related to patient selection, considering the short term of antibiotic treatment (14 days) and the high baseline scores of both NIH-CPSI and IPSS. In addition, no data regarding the different NIH-CPSI domains are reported [34]. Lastly, another study by Cai et al. involving two groups of patients treated with antibiotic alone and antibiotic plus Serenoa Repens, selenium, lycopene, bromelain and methylsulfonylmethane extract similarly reported an evident efficacy of nutraceutical products in addition to antibiotic therapy, in the treatment of chronic prostatitis [49]. Additionally, in this case, the difference with our study could be related to different baseline characteristics of patients involved as well as the natural compounds used. The present study has some strengths to be considered. First of all, this is the first study to evaluate the efficacy of Flogofilm^®^ in chronic BP treatment with the prospective design and the methodologic rigor. Despite this, there are some limitations that should be considered: the lack of a placebo, the relatively small sample size and the short follow-up time. In addition, the self-reporting nature of questionnaires could represent another potential bias, while the mixture of phytotherapeutics included in Flogofilm^®^ impede the evaluation of each compound alone. Further studies focusing on overcoming those limitations as the use of a double-blind and a placebo, in addition to a larger sample size, could represent an interesting perspective for future research.

## 5. Conclusions

The results of the present study suggest that the association of Flogofilm^®^ with fluoroquinolones for the treatment of patients affected by chronic bacterial prostatitis significantly improves pain, urinary symptoms and quality of life in both IPSS and NIH-CPSI scores compared with fluoroquinolones alone. Further double-blind placebo-controlled trials with larger sample sizes and longer follow-ups are needed to confirm these results.

## Figures and Tables

**Table 1:** Descriptive statistics of patients involved.

Variables	Group A	Group B	*p*-Value
Age (years)	34.62 ± 9.04	35.29 ± 10.32	0.755
PSA (ng/mL)	1.14 ± 0.67	1.25 ± 0.6	0.265
Prostate volume (mL)	31.51 ± 8.07	31.10 ± 8.73	0.962
Qmed (mL/s)	20.89 ± 1.52	21.41 ± 1.66	0.071
IPSS score	8.28 ± 6.33	9.88 ± 6.84	0.256
NIH-CPSI total score	21.70 ± 4.38	21.67 ± 6.06	0.959
NIH-CPSI pain domain score	9.62 ± 3.98	8.90 ± 5.18	0.265
NIH-CPSI urinary domain score	5.66 ± 1.82	6.08 ± 1.6	0.202
NIH-CPSI QoL domain score	6.43 ± 1.26	6.69 ± 2.07	0.201

**Table 2:** Comparison between the two groups at 1, 3 and 6 months after therapy.

		1 Month	*p*-Value	3 Months	*p*-Value	6 Months	*p*-Value
IPSS score	Group A	6.45 ± 4.8	0.020	5.32 ± 4.63	0.042	4.91 ± 4.47	0.005
	Group B	4.31 ± 4.35		3.20 ± 3.05		2.63 ± 3.28	
NIH-CPSI total score	Group A	16.15 ± 3.31	<0.0001	13.47 ± 3.07	<0.0001	9.83 ± 2.53	<0.0001
	Group B	13.10 ± 5.03		9.65 ± 4.23		5.51 ± 2.84	
NIH-CPSI pain domain score	Group A	5.83 ± 2.57	0.011	4.55 ± 2.54	<0.0001	3.09 ± 2.12	<0.0001
	Group B	5.04 ± 4.66		3.18 ± 4.13		1.31 ± 2.13	
NIH-CPSI urinary domain score	Group A	4.89 ± 1.38	<0.0001	4.23 ± 1.11	0.004	3.02 ± 1.03	0.005
	Group B	3.76 ± 1.33		3.51 ± 1.24		2.43 ± 1.06	
NIH-CPSI QoL domain score	Group A	4.89 ± 1.38	<0.0001	4.68 ± 0.98	<0.0001	3.72 ± 1.26	<0.0001
	Group B	3.76 ± 1.33		2.96 ± 1.24		1.78 ± 1.34	

## Data Availability

Data are available on request.
